# Sports and sustainable development: the troubling absence of meat sourcing policies in the sports sector

**DOI:** 10.3389/fspor.2024.1341810

**Published:** 2024-03-05

**Authors:** Chloe Sher, Caroline Fusco

**Affiliations:** Faculty of Kinesiology and Physical Education, University of Toronto, Toronto, ON, Canada

**Keywords:** sports, sustainable development, One Health, meat policy, industrial animal agriculture, antibiotic resistance, environmental pollution

## Abstract

The excessive use of antibiotics in industrial meat production in the U.S. incurs severe health implications for animals, humans, and the environment, thereby threatening the integrated health of the ecosystem and sustainable development. While the consumption of meat, including hot dogs, chicken wings, and hamburgers, is a hallmark of attending professional sports events in North America, the sourcing policies for meat in the realm of professional sports remain relatively obscure. We conducted a content analysis case study on the four major sports leagues in North America, their teams and stadium practices. Our objective was twofold: first, to investigate existing sustainability initiatives at the league, team, and stadium levels; and second, to examine whether there are any food sourcing programs, specifically meat sourcing policies that might encourage the consumption of meat produced without the use of antibiotics, in the sports sector that are designed to mitigate ecological ramifications of meat consumption within sports contexts. Results show that existing sustainability initiatives at the three levels are focused primarily on reducing carbon emissions and waste. There is, however, a notable neglect of food sourcing policies, which is concerning given that industrial animal agriculture is a leading cause of antibiotic resistance and environmental degradation. This suggests that meat sourcing policy is a missing piece in current sustainability initiatives. The major sports leagues should therefore consider incorporating pertinent policies, such as procuring meat-based products produced without the use of antibiotics to help strengthen their existing efforts in achieving their sustainable development goals.

## Introduction

Meat plays an integral part in shaping the distinctive food culture of sports, where iconic staples like hot dogs, chicken wings, and hamburgers consistently dominate the gastronomic landscape within stadia (1, 2). Indulging in these offerings has become a defining aspect of fan experience when attending a professional sports event in North America. Last year, during the Superbowl weekend in February, the National Chicken Association estimated that Americans devoured approximately 1.45 billion chicken wings (in stadia, in bars, at home) a record amount that is enough to circle the earth three times or equivalent to giving four chicken wings to every person in the United States (3). Compared to a decade ago, this year's number represents a 16% increase, and a 2% increase since last year (3, 4). In May, at a regular season Major League Baseball game between the Blue Jays and the Yankees, another record was set—fans in attendance consumed 61,111 hotdogs in a single game, which is equivalent to 1.7 hotdogs per fan, breaking the previous record by 18% (5, 6). The National Post wrote, “The hot dog-consuming madness at Blue Jays games is continuing at a record-breaking pace this year” (5). These examples demonstrate the staggering amount of meat products consumed during sports events and at sports stadia.

With increasing popularity in sports and fan base (7–10), demand for meat products (most likely industrially produced) will likely continue to soar in the sports sector. Food and quality of concessions at stadia are crucial to fans experience, the sport culture, and economics of sport (11, 12). Yet, apart from some attention paid to food safety at the stadium (13), scholars have not interrogated the sources of stadium foods, especially meat that is most likely rendered from industrial agricultural practices, which may have detrimental ecological impacts (14–17). Specifically, strong evidence suggests that the excessive use of antibiotics in meat production in the U.S. has led to the emergence of antibiotic-resistant bacteria and various forms of pollution, threatening the health of animals, humans, and the environment, and thus undermining the sustainable health of the planet (18).

While research on environmental sustainability in sports has increased notably in recent decades (19–23), few examine the current state of sustainability efforts among the largest sports leagues in North America (some notable exceptions are (24–26), or especially investigate how food policies and meat consumption in sport contexts seriously impacts planetary sustainability. To address this gap, we conduct a content analysis case study of the four major sports leagues in the U.S. to; (1) identify current sustainability initiatives led by each league, their clubs, and stadia, and (2) examine whether there are food policies to address the ecological impacts of meat consumption. We found that existing initiatives at the three levels primarily focus on mitigating carbon emissions and waste reduction. While some food programs exist, their primary emphasis is on produce. Consequently, the critical concerns surrounding the overuse of antibiotics in meat production and the development of pertinent meat sourcing policies have received limited attention within the four major sports leagues. While there has been a rise of critical animal studies in sport (27–29), only recently has literature on industrial animal agriculture's links to sport emerged (30, 31).

## Background

### Excessive antibiotic use in raising livestock

While the U.S. currently holds a prominent position as one of the world's leading meat producers, it also ranks as one of the largest global users of antibiotics in food animal production. Among developed, high-income countries, the U.S. stands out as the top consumer of antibiotics for raising food-producing animals (32). To put this into perspective, in 2020, U.S. producers used nearly twice as many antibiotics to raise livestock (170.8 mg/kg of livestock) compared to the Europe average (91.6 mg/kg of livestock) (33). Notably, beef producers in the U.S. surpassed all, employing three times more antibiotics to raise cattle (162 mg/kg of beef) than other major beef-producing countries in Europe, including the Netherlands (50 mg/kg), France (41 mg/kg), Denmark (32 mg/kg), and the United Kingdom (27 mg/kg) (34). This stark contrast in antibiotic use within livestock operations between Europe and the U.S. underscores the prevalent excessive use of antibiotics in U.S. meat production.

### The One Health approach

How we grow our food matters. Meat production practices not only can influence the quality of meat produced but also impose collateral costs on the entire ecosystem, jeopardizing its health and stability (14, 17). As the One Health approach underscores (35), animals, humans, and the natural environment are closely linked and interdependent on one another, forming an integrated ecosystem. Changes or disruptions in one facet of One Health, such as the excessive administration of antibiotics to livestock, not only will affect the health of animals but will set off a chain reaction affecting all entities within the ecosystem, including humans and the natural environment, ultimately causing irreversible damages to the ecosystem's delicate balance and sustainability (18, 35). As such, adopting the One Health approach is critical in addressing planetary health and sustainability (36).

In the following section, we highlight some key consequences, illustrating how the excessive use of antibiotics in U.S. livestock production not only detrimentally impacts animal welfare but also has far-reaching effects on humans and the natural environment. Hence, addressing antibiotic overuse in livestock farming is imperative for sustainable development. Adding meat sourcing policies to current sustainability initiatives by sports organizations can help strengthen their existing efforts in achieving sustainable goals.

### Animal health implications

To begin, it is crucial to recognize that the excessive use of antibiotics in U.S. livestock production is largely due to poor living conditions many animals endure (37–39). Animals such as chickens, pigs, and cattle often suffer due to substandard production conditions, necessitating antibiotic intervention for increased survival rates and production yields (38, 40, 41).

Livestock often lives in overcrowding and unhygienic living conditions, rendering them susceptible to illnesses (37–39). Chickens, for example, are prone to developing chronic respiratory diseases (CRD) due to high levels of ammonia in their confined and overcrowded living spaces. These birds often live atop composted litter, with their living quarters seldom cleaned of accumulated droppings. This leads to the build-up of ammonia, which can damage the mucous membrane in their airways, making them vulnerable to respiratory infections (37). Pigs, similarly, frequently suffer from swine dysentery (an infectious disease marked by severe diarrhoea and inflammation of the large intestine) and swine arthritis, primarily due to overcrowded and unsanitary pens (38, 39).

Livestock are often fed manipulated diets designed to accelerate growth, making them vulnerable to getting sick. Cattle, for instance, often develop liver abscesses because they are fed an intense, highly enriched grain-based diets aimed at rapid weight gain before slaughter (34, 40, 42). These diseases are common and can be fatal, thereby reducing production yields. As a quick fix, livestock producers typically administer antibiotics to livestock routinely, even when they are healthy, to help compensate for substandard conditions as well as minimize the side effects of manipulated feed.

A key contributing factor to the excessive antibiotic usage in the U.S. is the lack of government regulations. Currently, livestock producers in the U.S. are permitted to use antibiotics for preventative purposes, provided they have a veterinarian's approval—a practice that has been banned in the European Union (EU) (43). EU regulations state that antibiotics shall not be used to prevent diseases before animals get sick or treat groups of animals where some might already be sick. In other words, antibiotics may be administered only to sick, individual animals (and not whole herds) (43). Therefore, unlike the U.S., the EU has strict regulations to prohibit livestock producers from using antibiotics to compensate for low welfare practices.

Amidst lax regulation in the U.S., many medically significant antibiotics can be purchased as over-the-counter products with minimal oversight regarding dosage and duration of use (44, 45). The lack of government regulations on antibiotic use in livestock operation hence fosters poor livestock welfare, as antibiotics remain easily accessible, discouraging livestock producers from implementing improved livestock farming practices such as sanitization and feed quality, which would reduce animals' susceptibility to illnesses and suffering.

One significant consequence of the excessive use of antibiotics in livestock is the emergence of antibiotic-resistant strains in animals (46). Bacteria live in the guts of the animals (47). When animals receive constant doses of antibiotics, it may lead to the emergence of antibiotic-resistant strain in their internal system, making infection hard to treat with common antibiotics. Take cattle as an example: The U.S. Food and Drug Administration (FDA) found that Enterococcal species, prevalent in cattle's ceca, increased resistance to macrolides, one of the most consumed antibiotic classes in cattle operation in the U.S (47). Data from the FDA indicates that resistance to tylosin, a macrolide antibiotic, nearly tripled from 6.3% in 2013 to 18% in 2017. While data beyond 2017 remains unavailable for reasons unknown, it is highly probable that this trend has persisted, given the continued increase in tylosin use within U.S. cattle production (48–51). This suggests that the consistently high level of antibiotic use in the U.S. for raising livestock is facilitating the rapid development of antibiotic-resistant bacteria in animals, compromising their health and well-being.

In sum, the substantial antibiotic consumption in U.S. livestock production not only reflects poor livestock production practices and human disregard for animal well-being (43) but also accelerates the development of antibiotic-resistant bacteria (47), posing significant health implications for animals and other entities (e.g., humans) in the ecosystem, including professional sports.

### Human health implications

The proliferation of antibiotic-resistant bacteria in animals carries profound consequences for human and animal health. These bacteria may often infect humans and cause infections that are hard to treat by most available first-line antibiotics, resulting in increased mortality (52). This is largely because the majority of antibiotic used in livestock production are the same classes of antibiotic as those used in human medicine (48, 50). A recent report from the U.S. FDA estimated that two-thirds of antibiotics considered medically important for humans sold in the U.S. are administered to farmed animals (48, 53). Another FDA report shows that seven out of nine antibiotics classified as medically important to human medicine experienced an increase in usage from 2020 to 2021 (48). As a result, the overuse of antibiotics in livestock farming is rapidly eroding the effectiveness of available antibiotics in treating humans' infections.

Consider, for instance, the cases of macrolides and tetracyclines, both crucial for human health. Macrolides and tetracyclines are prescribed for treating bacterial infections like pneumonia, sexually transmitted diseases, and infections of skin, respiratory tract, intestinal, urinary tract, etc (54, 55). As such, both antibiotic classes are classified as “highest priority critically important” and “highly important” for human medicine by the World Health Organization (WHO), respectively (56). “Highest priority critically important” means that the antibiotic class is either the sole or one of the very few treatment options used frequently to treat people with serious bacterial infections caused by antibiotic-resistant bacteria from non-human sources (56). Similarly, the U.S. FDA, utilizing a less thorough binary classification system, also classified both antibiotic classes as “medically important” (49).

Despite their significance in human medicine, macrolides (e.g., tylosin) and tetracyclines (e.g., oxytetracycline) are the two most used antibiotics in U.S. livestock farming (48). They are administered to livestock to prevent diseases such as liver abscesses in cows, respiratory diseases in chicken, and arthritis and dysentery in pigs (38, 40, 41). Currently, both are approved by the FDA for over-the-counter use in food-producing animals (44, 45). With minimal regulations, macrolide use surged by 21% from 2020 to 2021, while tetracycline use remained persistently high (48, 49). In fact, a study in the U.S. revealed that cattle were treated with macrolide antibiotics for an average of 132 consecutive days (57), highlighting the injudicious use of antibiotics critical for human health in livestock production. The hyper-consumption of animal products at sports stadia daily during professional sports seasons reproduces this overuse and exacerbates its impacts on human health because of meat supply chains.

Below, we highlight two prevalent mechanisms that demonstrate how the excessive use of antibiotics in livestock farming in the U.S. may impact human health.

#### Foodborne illnesses

Antibiotic-resistant bacteria originating in animal guts often find their way into human populations through foodborne transmission, particularly via undercooked meat or contact with contaminated meat products (58). Bacteria like Salmonella and Campylobacter, responsible for two leading foodborne diseases in the U.S., have developed resistance to many first-line antibiotics due to antibiotic overuse in livestock farming, leading to a concerning rise in antibiotic-resistant infections (52). The latest data from the U.S. Centers for Disease Control and Prevention (CDC) displays an alarming pattern, showing a 44% rise in antibiotic-resistant Campylobacter infections since 2013, totaling over 448,000 cases in 2019 (52). Similarly, antibiotic-resistant nontyphoidal Salmonella cases reached 212,500 in 2019, representing a 113% increase since 2013 (52).

The injudicious use of antibiotics in livestock farming has significant implications for food safety, as these antibiotics can be absorbed into animal tissues. Researchers have found residues of commonly used antibiotics (e.g., tylosin and tetracycline) in retail meat, including in muscles, fat, and liver tissues (59, 60). This raises concerns about consumption safety and potential health implications (60–62). Moreover, it brings to our attention the possibility that these kinds of antibiotics may be present in meat supply chains to sports organizations. Not only would this be detrimental to human health but, as stated earlier, it has unmeasured detrimental environmental impacts that can be traced back to the industrial agricultural system (63, 64).

#### MRSA

Beyond foodborne illnesses, the excessive use of antibiotics in animals raised and slaughtered for food has given rise to highly antibiotic-resistant strains of methicillin-resistant Staphylococcus aureus (MRSA), a type of bacteria capable of causing deadly skin, blood, and lung infections (65). In the past, MRSA was primarily associated with healthcare settings (e.g., hospitals), known as healthcare-associated MRSA or HA-MRSA. However, in the last decade, infections known as livestock-associated MRSA or LA-MRSA, have been acquired outside of healthcare settings, including livestock farms. While LA-MRSA initially infected only animals, evidence suggests that the excessive antibiotic use in food animals has allowed LA-MRSA to evolve, becoming capable of infecting humans (66, 67) through various pathways, including direct contact with animals and contaminated meat, exposure to MRSA-contaminated dust, and human-to-human transmission (68–71). A systematic review reveals that livestock workers are particularly at risk of LA-MRSA infection (68).

One prominent strain of LA-MRSA that has emerged to infect humans is MRSA ST 398. In the U.S., Smith et al. (72) first reported its presence in both pigs and pig workers in the U.S. Midwest region. In a study involving 299 animals and 20 workers in two pig production pyramids in Iowa and Illinois, they found that 49% of the animals and 45% of the workers were colonized with MRSA ST 398. Although LA-MRSA infections in humans are less common than HA-MRSA and constitute a smaller proportion of all MRSA cases, the rising incidence is a cause for significant public health concern (66, 73, 74). Given the association of meat consumption with sports—Young (2014) has called sports “a carnist culture”— sports stadia may be a significant site for the transmission of some of these antibiotic-resistant strains.

### Environmental impacts

Antibiotic-resistant bacteria developed in animals due to industrial animal agriculture can spread and contaminate the natural environment, including air, soil, and water resources, through runoffs and improper manure disposal practices (75). This often leads to many forms of pollution in communities near livestock operations and contributes to the development of antibiotic-resistant bacteria in the environment (75, 76). Importantly, once the natural environment is contaminated with antibiotic-resistant bacteria, any species that come into contact will also be infected, triggering a series of contaminations within the ecosystem (77, 78).

#### Air pollution

Pig operations, for example, are notorious for causing air pollution in nearby communities due to their manure disposal practices. Pig manure is typically stored in open lagoons, often referred to as cesspools. To prevent these cesspools from overflowing, liquified waste is sprayed onto nearby fields using large, sprinkle-like apparatuses, thereby releasing antibiotic-resistant bacteria and other pollutants into the air (79, 80). Research indicates that LA-MRSA can be carried up to 300 m away from industrial pig operations through airborne transmission (81). Furthermore, studies consistently report elevated levels of ammonia (82), endotoxin (83), hydrogen sulfide (84) and particulate matter (PM) (85) in the air around pig operations.

When the air is contaminated, it can, in turn, affect the wellbeing and health of plant life and multiple species in the surrounding communities, including humans (79, 80). For example, studies show that contaminated air can adversely affect the health of nearby residents, resulting in higher rates of nausea and respiratory issues, including breathing difficulties, wheezing, asthma, and high blood pressure (86, 87). Additionally, CAFO's (Controlled Animal Feeding Operations), particularly in the United States are more likely to be in areas with a high percentage of racial and ethnic minorities bringing to our attention the environmental injustices and racism connected to industrialized meat production (87–91).

#### Soil contamination

Soil contamination also arises when manure from antibiotic-treated animals is used as fertilizer. Manure serves as a reservoir for antibiotics and antibiotic-resistant bacteria. When applied to agricultural soil, it leads to the dissemination and proliferation of antibiotic resistance genes, the selection of resistant bacterial populations in soil, and disruptions in the composition of soil bacteria (17). Onan and LaPara (92), for example, found that subtherapeutic antibiotic use (before animal becomes ill) in animal farming substantially increases the concentration of antibiotic-resistance bacteria in manure and nearby soil. They found that the proportion of antibiotic-resistant bacteria to tylosin (a common antibiotic in animal husbandry) in manures from cattle and pigs who received antibiotics was as high as 25.8% and 69%, respectively (92). In contrast, the proportion was lower than 3.3% in manure from dairy cows, where antibiotics were used exclusively for specific therapeutic purposes (i.e., treating sick animals) (92). Numerous studies consistently find significantly higher levels of antibiotic resistance bacteria in soil fertilized with animal manure compared to unmanured soil due to the use of antibiotics in animal husbandry (75, 92, 93).

Tainted soil poses heightened risks to humans by increasing the likelihood of antibiotic-resistant infections, such as MRSA. A study conducted in Pennsylvania showed that individuals residing near high-intensity livestock operations and crop fields fertilized with pig manure had over a 30% higher likelihood of developing MRSA infections compared to those with the lowest exposure levels (94). Furthermore, research indicates that when antibiotic-resistant bacteria from manure disrupt soil microbial communities, it can profoundly alter soil functions and diminish its capacity to sequester carbon (17). Thus, the effects of meat production for any aspect of human consumption (i.e., individual, family, sports events etc.) are profoundly interconnected with environmental degradation (63, 64).

#### Water contamination

Antibiotic resistance genes and bacteria in manure often leach into aquatic ecosystems via runoff, thereby disrupting the microbial balance within water bodies (14). For instance, a study in the Chesapeake Bay region found that streams near areas with denser poultry barns had a higher concentration of E. coli isolates resistant to multiple antibiotic classes compared to streams near areas with fewer poultry barns (14). This demonstrates poultry operations' role in disseminating antibiotic-resistant bacteria into water environments, with implications for the well-being of aquatic species (14, 95).

Furthermore, manure contains high levels of nutrients, including nitrogen and phosphates. When these nutrients enter waterways, they can cause harmful algae blooms, suffocating aquatic species and disrupting aquatic ecosystems. The chicken industry, for instance, discharges approximately 12 million pounds of nitrogen into the Chesapeake Bay through runoff each year (76). As a result, nearly 78% of the Chesapeake Bay has been impaired by toxic contaminants since 2020 (95). This highlights the potential for livestock production to cause widespread water contamination and disrupt the balance of aquatic ecosystems if proper manure management to prevent runoff is not implemented (76).

Water is essential to all living organisms on our planet. When water is polluted, it can rapidly transfer the bacteria to other entities within the ecosystem and set off a chain reaction within the food chain (78). One prime example is contaminated water used for crop irrigation. E. coli O157:H7, for instance, is a highly pathogenic class of bacteria that are resistant to multiple antibiotics and typically found in the gastrointestinal tracts and feces of cattle (96). In humans, it can induce severe intestinal infections by producing a potent toxin that damages the lining of the intestinal wall, leading to symptoms like bloody diarrhea. Children are particularly vulnerable, as the infection can result in acute kidney failure. A deadly outbreak in 1993, in which four children lost their lives and 732 people fell ill due to E. coli O157:H7 contaminated ground beef led the Department of Agriculture's (USDA) Food Safety and Inspection Service (FSIS) to declare E. coli O157:H7 an adulterant in ground beef in 1994. This meant that raw ground beef for sale could not contain any trace of E. coli O517:H7, as a measure to protect consumers (97). Subsequently, fewer reported cases of E. coli O157:H7 infections linked to beef consumption were reported (98).

However, in recent years, E. coli O517:H7 found another route to infect humans—through leafy greens such as lettuce and spinach [e.g. (78, 99),]. Investigations found that contaminated products often originated from farms near cattle feedlots (e.g., farms in Yuma, Arizona (77) and Solinas, California (78). It is believed that E. coli O517:H7 from cattle may have leached into the water canals used for crop irrigation, subsequently transferring the bacteria to leafy greens, and causing human infections.

Finally, researchers have also identified that soil and water tainted with bacteria can facilitate the transmission of E. coli O517:H7 through the root systems and into the internal tissues of plants (100). This suggest that chemical disinfection of the outer surface of the vegetable may not be effective in removing the bacteria. Indeed, most recent data from the Interagency Food Safety Analytics Collaboration (IFSAC) shows that over 55% of E. coli O157 illnesses were attributed to vegetable row crops, such as leafy greens, while beef products only account for only 23% (98). This shows that once antibiotic resistant bacteria in animals are leached into the water and natural environment, it can set off a chain reaction within the ecosystem, affecting humans and nonhumans species that come into contact with the pollutants. Sports stadia also use vegetables (most likely from mass row crop farms) in their menus along with meat (e.g., lettuce on a hamburger). It is not unlikely, then, that an interrelation between what is consumed at professional sports stadia, industrial animal and vegetable agriculture, water, and soil contamination, as well as air pollution, exists.

### A public health crisis

The far-reaching consequences of the excessive use of antibiotic in livestock farming has triggered a public health crisis. In the U.S. alone, a recent CDC report shows that antibiotic resistance alone is responsible for over 35,000 annual deaths, with 2.8 million individuals developing drug-resistant infections each year (52). The WHO has indeed declared antimicrobial resistance as “one of the top 10 global public health threats facing humanity” (101) and vigorously advocates for global communities to cease the use of antibiotics in healthy animals for livestock production (18). Likewise, the United Nations calls the antimicrobial resistance a crisis that “threatens the achievement of the Sustainable Development Goals” and constitutes “a fundamental threat to human health, development and security” (102).

### One Health perspective

The One Health approach asserts that a healthy and sustainable planet depends on the well-being of animals, humans, and the natural environment (35). Thus, to enhance the planet's sustainability, the health of all three must be simultaneously safeguarded. Actions that disrupt the One Health equilibrium, such as the excessive use of antibiotics in livestock production, must be curtailed and discouraged.

As the evidence indicates, the excessive use of antibiotic by U.S. livestock producers is disrupting the One Health balance, initially affecting animals' wellbeing, and subsequently impacting humans and the natural environment. Additionally, once these entities are affected, they, in turn, affect other species that come into contact, triggering further exponential consequences within the ecosystem, and undermining the sustainable health of the planet (35).

As we have stated previously, given the significant quantity of meat products consumed at sports events, there is an urgent need to require meat sourcing policies to address antibiotics overuse in livestock production for meat that is likely sold and consumed in sports facilities.

### International governing bodies, mega events, and food sourcing policies

Currently, among international organizations, the International Olympic Committee (IOC) is the only organization that has published food sourcing recommendations for the sports sector. However, it is important to note that the current guidelines provided by the IOC pertain exclusively to produce (e.g., vegetables) and do not include non-produce items, such as meat products. Furthermore, the primary focus of the IOC's food program is centered on addressing carbon emissions rather than the sustainability costs associated with meat production practices.

Specifically, within its “Sustainability Essentials” guides, IOC urges organizations to reduce the carbon footprints of their food services by implementing the following measures: (1) sourcing produce produced locally and from environmentally responsible agriculture, (2) reducing food waste through redistributing surplus food, and (3) using sustainable food packaging (103).

In contrast, mega-events have taken a more comprehensive approach to food sourcing, which includes meat products. During the London 2012 Olympic Games, for example, the organizers adopted the “London 2012 Food Vision,” a strategic initiative that underscored how sustainable food procurement policies can contribute to a healthier and more environmentally conscious event (104). The Games' organizers considered the origin and production methods of the food, with the goal of ensuring that all food served at the games met high standards for quality, safety, and sustainability (104). The plan outlined detailed sourcing standards for a diverse range of food items, such as meat products, produce, dairy, eggs, and seafood.

Similarly, the forthcoming Paris 2024 Olympic Games have also introduced a comprehensive food strategic plan, known as the “Paris 2024 Food Vision,” aimed at responsibly sourcing a wide array of food items to promote sustainability through smart catering choices (105). Regarding meat products, the plan explicitly states that all meat will be sourced from France and certified with a specific label.

While both food visions do not explicitly mention concerns related to antibiotics in meat products, both France and Britain do adhere to strict EU regulations on antibiotic use in the raising of animals for food and have some of the lowest antibiotic usage rates worldwide (106). Nevertheless, these examples show that meat sourcing programs have been considered to an extent and are being implemented in mega-events, like the Olympic Games. They also demonstrate that there is increasing recognition of the pivotal role that sustainable foods can play in fostering greener sports events and contribute to sustainable development.

### Sustainability movement in major sports leagues

In recent decades, major sports organizations and teams in the U.S. have intensified their efforts to promote environmental sustainability and contribute to sustainable development (107–109). This growing commitment to sustainability within sports organizations reflects the increasing recognition of the threats posed by environmental degradation to the sports industry. Gary Bettman, the NHL's commissioner, highlighted the importance of this cause in 2014 when he stated, “We have a vested interest in this cause. As a business, we rely on freshwater to make our ice, on energy to fuel our operations, and on healthy communities for our athletes, employees, and fans to live, work, and play” (110).

Since the 2000s, the four major sports leagues have actively collaborated with environmental organizations to advance their environmental initiatives. For instance, MLB (2006), NBA (2007), NFL (2008), and NHL (2008), as well as MLS (2009), joined forces with the Natural Resources Defense Council (NRDC), one of the first non-profit environmental organizations founded in 1970. Sports partnerships with the NRDC were initiated to seek expert guidance on environmentally sustainable practices within the realm of sports (111, 112). Additionally, since 2010, these leagues have forged partnerships with the Green Sports Alliance (GSA), a non-profit organization dedicated to leveraging the influence of sports to foster sustainable communities (113). The impact of these efforts was particularly highlighted in 2016 when the GSA and the NHL joined forces with President Barack Obama to designate October 6th as the inaugural Green Sports Day.

Despite notable progress in advancing sustainability within the sports realm, it should be noted that scholars have pointed to potential problems of “greenwashing” (114–116). Sports event organizers, facilities, and organizations, who often adopt a market-oriented approach to environmentalism, tend to promise sustainable commitments, operation practices, and goals for marketing purposes, but in reality, fail to fulfil these promises.

While sustainability efforts in the four major leagues have made substantial strides, there appears to be a dearth of action addressing sustainable food choices. As an example, on Earth Day 2020, MLB proudly unveiled a list of 100 green initiatives but only one initiative was related to sustainable food (item#92) (117). This raises questions about the implementation of food sourcing programs within the four major leagues and the extent to which sustainable food programs have been integrated into their sustainability agendas. Further examination regarding the incorporation of sustainable food options within these leagues is therefore warranted.

### Sport environmentalism studies

In the early literature exploring the intersection of sports and environmental sustainability, a vital aspect discussed was the significance of food sourcing. One of the first authors in the field, David Chernushenko, who authored “Greening Our Game” in 1994, drew attention to this issue. Chernushenko, an early advocate for environmentalism in sports, recognized the link between major sports events and their environmental impacts. He provided a set of recommendations for fostering a socially and environmentally responsible sports industry. Within the context of food services at events and facilities, Chernushenko (118) underscored the importance not only of environmentally friendly food packaging but also of offering organically grown, chemical-free food. He stated, “What food is served in or on is important, but equally so is the type of food itself. Where does it come from? How was it grown? And what was it treated with?… It seems paradoxical that extraordinary measures are taken to protect athletes from intentional food poisoning, while institutionalized poisoning of food through chemical treatment is entirely ignored” [(118): 190–191].

However, despite Chernushenko's early concern for food production practices, this aspect continued to be neglected within the sports industry and in literature. While research on environmental sustainability in sports has notably expanded in recent decades, with the establishment of a subdiscipline called “sport ecology” (21), few studies have delved into how food sourcing practices in sports contexts, such as stadia, can negatively impact sustainable development due to irresponsible production practices, including the excessive use of antibiotics. For example, an important and recent contribution to the intersections of stadia and environmental justice (119), appears to have no references to animal agriculture, food, or meat consumption and their relationship to stadium sustainability in the edited collection.

When examining the impact of sports on the environment and sustainability development, scholars have focused on how the hosting mega-events like the Olympic Games (120, 122) can often damage the natural habitat through the construction of sports facilities for various disciplines (e.g., alpine skiing (118, 122), sledding (123), and golf (124, 125). Another area of examination has been the environmental impact of the consumption culture in sports, particularly concerning the carbon footprints associated with the production of sports goods and food services. For instance, Szto and Wilson (126) argue for reducing the carbon footprints of bike production, advocating for more environmentally friendly production practices and recycling options for bicycles. The International Olympic Committee (IOC) encourages organizations to minimize the carbon footprints of food services by reducing food waste through redistributing surplus food and utilizing sustainable food packaging, given the substantial consumption and waste generated during sporting events (103).

To date, the issue of sourcing meats produced without the use of antibiotics, and the subsequent contamination and pollution that stems from industrial animal agriculture, within the sports context, has received scant attention in literature. Notably, only two sports study addresses this matter. Heinze and Soderstrom (127), in their examination of sustainability practices in stadia, identified three pivotal areas for venues to prioritize: sourcing, energy and environmental design, and waste management. Within the realm of sourcing, they provided a set of considerations, which includes “choosing meats produced without the use of antibiotics” (p. 269). Henly and Krenza (128) discuss sustainable food options in stadia and suggest food procurement policies should target meat products produced without the use of antibiotics.

Choosing meats without the use of antibiotics of course is not a panacea, while animals may be raised in more healthy conditions, there are still concerns about, for example, “grass-fed beef” (129, 130) and whether it is better for the environment. And, of course, however animals are raised, ethically, for many, it remains highly problematic that; i) billions of animals are bred into existence through industrial animal agricultural systems that exploit, and subject them to some of the most egregious socially sanctioned human violence throughout their lives and when they are slaughtered (131–134), and ii) that those systems and their processes cause massive amounts of environmental degradation (16, 136, 137). Preferably for sustainability and ethical goals, sports would not be so intertwined with industrial animal agricultural systems in the future, which might allow sports to reach those (un)attainable sustainability goals.

Taken together, the profound implications of consuming meat products derived from the excessive use of antibiotics, and producing contamination and pollution, in sports contexts have remained a relatively neglected topic in the literature. Information about the extent to which food and meat sourcing policies are currently put into practice among sports leagues, their affiliated clubs, and the stadia across the U.S. will help provide a clearer picture of this vital aspect of sports sustainability and inform policy making to help advance sustainable development.

## Method

We conducted a case study on the four major sports leagues (the NFL, MLB, NBA, and NHL), their teams, and stadia to examine current sustainability initiatives implemented at the three levels and to find out whether there are food sustainability policies. These four leagues are currently the largest in North America with a combined revenue of over $38.1 billion in 2022 (Table 1).

**Table 1:** Total revenue earned by the four major leagues in 2022.

League	Revenue in 2022 (USD)
NFL	$12 billion [Ozanian, (138)]
MLB	$10.8 billion [Anderson, (139)]
NBA	$10 billion [Forbes, (140)]
NHL	$5.3 billion [Shapiro, (141)]
Total:	$38.1 billion

We conducted the analysis at three separate levels for several reasons. First, the leagues, teams and stadia are often owned by different entities. This can influence control over sustainability programs. Second, analyzing at three levels allows us to examine the relationship between the league and their teams. For example, whether league-driven initiatives may have bottom-down effects and influence their teams to take “green” actions. Third, it allows us to investigate the relationship between teams and their home stadia. Some stadia are owned by the same people who own the team, suggesting that the team likely have direct control over food options in the stadium. On the other hand, teams who play at a stadium owned by a third party may not have direct control over the stadium management. Thus, there is a need to separate the analysis at the team and stadium level.

In terms of teams, since there are many teams across North America, we focused our attention on teams in three cities—Los Angeles, Chicago, and New York City. These cities are the most populated on the West Coast, Midwest, and East Coast, respectively. They also have teams in all four major sports leagues. In some of these cities, there is more than one team in each league. New York City, for example, has three NHL teams. In such cases, for analysis purposes, we examined only the top-performing team based on the latest standings in the league. We focused only on the better-performing teams because they often receive greater media exposure, have a bigger fan base, operating budgets and larger scales of operation, and hence greater social influence on sustainability causes. We examined a total of 12 teams, four teams representing the four leagues in each city (Table 2).

**Table 2:** Teams from the four major leagues that are included in the analysis.

	NFL	MLB	NBA	NHL
Los Angeles, California	• Los Angeles Chargers • Los Angeles Rams^a^	• Los Angeles Dodgers • Los Angeles Angels^a^	• Los Angeles Clippers • Los Angeles Lakers^a^	• Los Angeles Kings • Anaheim Ducks^a^
Chicago, Illinois	• Chicago Bears	• Chicago Cubs • Chicago White Sox^a^	• Chicago Bulls	• Chicago Blackhawks
New York City, New York	• New York Giants • New York Jets^a^	• New York Yankees • New York Mets^a^	• New York Knicks • Brooklyn Nets^a^	• New Jersey Devils • New York Rangers^a^ • New York Islanders^a^

^a^ The team was excluded from the analysis due to lower standing as of June 15, 2023 (142–145).

Lastly, we focused on stadia that are home to the selected teams since stadia are key sites of consumption. The stadia included in the analysis are shown in Table 3.

**Table 3:** Stadia that are included in the analysis.

	NFL			NBA		
**Team**	NY Giants	Chicago Bears	LA Chargers	New York Knicks	Chicago Bulls	Los Angeles Clippers
**Stadium**	MetLife Stadium	Soldier Field	SoFi Stadium	Madison Square Garden	United Center	Crypto.com Arena
	MLB			NHL		
**Team**	NY Yankees	Chicago Cubs	LA Dodgers	NJ Devils	Chicago Blackhawks	LA Kings
**Stadium**	Yankee Stadium	Wrigley Field	Dodger Stadium	Prudential Center	United Center	Crypto.com Arena

To collect the most up-to-date and credible data, we scoured the official websites of the leagues, the teams, and the stadia. Most leagues, teams and stadia have a dedicated webpage on sustainability commitments to showcase their green efforts. The search was conducted in March 2023.

## Results

We first examined green initiatives by the four major sports leagues. We found that each of the leagues has established a “green” program to illustrate their commitments to sustainability. NFL Green was the first to be established in 1993, followed by NBA Green and MLB Green in 2008, and NHL Green in 2010. Based on their mission statements, their sustainability goals are similar in two ways. First, three of the four leagues (except the NBA) aim to take responsible actions for the environment. They used the words “responsible steward” and “responsible and socially conscious sustainability efforts”. Second, three of the four leagues (except MLB) emphasize on forming partnerships with teams, fans, players, and/or employees to promote green efforts. Except for the NFL, all leagues have listed numerous environmental partners. Bonneville Environmental Foundation and the Green Sport Alliance (GSA), for example, are listed in the three league sites. Yet there is limited information provided about the types of programs involved in these partnerships.

In terms of concrete sustainability programs implemented by the four leagues, all leagues promote green practices at their signature events. The NFL, for example, has a Super Bowl environmental program to reduce waste and carbon emissions. Similarly, MLB also collaborate with clubs to promote sustainability efforts during the All-Star Week. The NBA promoted electric vehicles and used hybrid buses to shuttle fans to NBA All-Star events. The NHL purchased carbon offsets to counter Stanley Cup Playoff air travel in 2019.

Compared between the four leagues, it is noteworthy to point out that some leagues have unique initiatives to encourage their teams to adopt green practices. MLB, for example, presented the “Green Glove Award” (since 2008) to the club with the highest recycling rate. Similarly, NHL hosts the NHL Green Week (since 2016) which encourages clubs to take part in a green project to reduce environmental impacts. On the other hand, the NFL and the NBA show their sustainability commitment by signing the United Nations' Sports for Climate Action Framework.

Existing sustainability initiatives by all four leagues primarily focused on two fronts—reducing carbon emissions and waste. To reduce carbon emissions, their actions can be categorized into three types—purchasing carbon offsets, planting trees in local communities, and promoting green transportation. To reduce waste, they focus on reducing the use of plastic containers, recycling, and donating unused food. MLB and the NHL also reported water conservation actions through purchasing water restoration credits.

Focusing on food, MLB was the only league that included food sourcing information in its sustainability programs. They report that 11 ballparks operate on-site gardens to provide fresh produce. Overall, at the league level, there are very few food programs, and they focus on produce only (Table 4).

**Table 4:** Sustainability programs implemented by the NFL, NBA, MLB, and NHL.

	NFL	NBA	MLB	NHL
Green initiative	NFL Green (since 1993)	NBA Green (since 2008)	MLB Green (since 2008)	NHL Green (since 2010)
Mission statement	“The NFL looks to be a responsible steward of the environment in all areas of its business -- using resources efficiently and minimizing waste. The focus of the NFL's environmental efforts is on greening NFL events and facilities, and working with NFL teams to help them operate their businesses in sustainable, eco-friendly ways.”	“The NBA is committed to promoting environmental sustainability in the communities where we live, learn and play. Through NBA Green and the power of basketball, the NBA will continue to inspire our fans and partners to minimize environmental impacts and help activate broader industry and societal progress through our actions, transparency, education, and engagement.”	“Major League Clubs emphasize and practice sustainability through waste diversion, composting, sustainable purchase, water conservation and energy efficient practices throughout the year. All of Major League Baseball is committed to practicing responsible and socially-conscious sustainability efforts. Key initiatives include Green Team activations, front office volunteer efforts, food donations to local charities and substantive programs operated by MLB Clubs.”	“The National Hockey League is committed to promoting sustainable business practices with Member Clubs, players, fans, employees and partners, so all can be more responsible stewards of the planet by reducing the use of resources, and reusing and recycling equipment and materials.”
Environmental program	– Signed UN's Sports for Climate Action Framework – Super Bowl environmental program for over 15 years. In each Super Bowl host community, the NFL works with local partners to develop: solid waste management, material reuse, food recovery, sports equipment and book donations, and greenhouse gas reduction.	– Signed UN's Sports for Climate Action Framework – NBA League Offices in New York City and Toronto have received LEED (Leadership in Energy and Environmental Design) Certification and LEEDCore+Shell Platinum Certification, respectively.	– “Green Glove Award” presented to the club with the highest rate of waste-diversion – Collaborate with clubs during All-Star Week to promote sustainability efforts – Greening efforts are broadcasted to fans at the ballpark during Postseason – all of its Clubs are members of the Green Sports Alliance	– NHL Green Week, a week dedicated to educating and engaging fans around environmental concerns and the environmental efforts of the League and its Clubs – 2016, NHL Greener Rinks Initiative™ launched. – NHL became a founding member of Sport and Sustainability International (SandSI), an organization committed to advancing the UN Sustainability Development Goals, the goals of the Paris Agreement, and the UNFCCC. – Partnered with President Obama to launch the first Green Sports Day (Oct 6)
Carbon reduction	Carbon offset – Green Energy: purchasing renewable energy certificates to offset energy used at event facilities. Tree planting – Partnered with local communities and recreation department to develop community greening projects including planting trees and creating community gardens. – Planted 57 trees in Arizona last October in honor of Super Bowl in 2023 Natural habitat building – Coral reef restoration project "100 Yards of Hope" in partnership with Miami Super Bowl host in 2020.	Tree planting – To celebrate Arbor Day in April, donated 75 trees for every 3-pointer shot made in games between April 29-May 4 which resulted in 25,000 trees planted in Saharan Africa. Transportation – Promote electric vehicles and used hybrid buses to shuttle fans to NBA All-Star events. Education – Created the NBA’s "Carbon Footprint Quiz" which helps fans learn ways to minimize carbon footprint	Carbon offset – MLB offsets all carbon emission associated with ballpark energy used and players' travel during the All-Star Week through buying renewable energy credits. Energy use – Installed motion-sensored LED lighting and an updated television schedule to reduce energy consumption at league's office. Transportation – Encourage fans to take different forms of transportation by providing walking paths between popular locations throughout All-Star Week	Carbon offset – NHL to purchase carbon offsets to counter Stanley Cup Playoff air travel in 2019. _Hold Green awareness month (2014, 2018 report available)
Waste reduction	Recycle – Partner with Verizon to recycle e-waste before Super Bowl. – Work with host stadium to increase recycling and waste diversion rate. Reduce – Donate more than 140,000 pounds of donatable food and beverages from Super Bowl to local food banks. – Donate event materials (e.g., décor fabric, carpet, building materials) to Habitat for Humanity, Salvation Army etc.	Reduce – Reduced the use of plastic bottles by 80% in league's offices.	Recycle – Collaborate with Clubs and Green Teams to collect recyclables in between innings during Postseason. – Collaborate with TerraCycle to recycle disposable masks, gloves, gowns, etc. Reduce – eliminated paper plates and plastic cups at the league's offices. _Supply refillable water bottles during All-Star Week.	Reuse – partner with Adidas that all NHL teams wore Adidas Primegreen jerseys (partially made of recycled plastic) during 2021-2022 season. Upcycle – Rink2Reef program where broken hockey sticks are upcycled to create marine habitats in local waterways.
Water conservation			Water credit – MLB offset water used at the All-Star host ball park through purchasing renewable energy credits	Water credits – purchase of water restoration credits in partnership with our partner Bonneville Environmental Foundation (BEF) – NHL's Water Neutral Stanley Cup Final – "Gallons for Goals" program where NHL pledged to restore 1,000 gallons of water to a river with critically low flow for every goals scored during regular season.
Food sourcing			On-site garden – 11 Ballparks operate on-site gardens to provide fresh produce	
Green Partner	Verizon, local non-profit organizations (not specified)	-Arbor Day Foundation – Bonneville Environmental Foundation – Clever Carbon – Green Sports Alliance – National Environmental Education Foundation – South Pole – Sport and Sustainability International	– Bonneville Environmental Foundation – Council for Responsible Sport – Green Sports Alliance – Measurabl – Priceless Planet Coalition	– Sport and sustainability international – Green Sports Alliance – Fastenal – Opteon Refrigerants – Signify – Bonneville Environmental Foundation
References	NFL. (2023). NFL Green. https://www.nfl.com/causes/nfl-green/	NBA. (2023). NBA Green. https://cares.nba.com/programs/nba-green/ NBA. (2023). Team arenas. https://green.nba.com/teams-arenas/ NBA. (2023). NBA Green: Dr. Allen Hershkowitz leads sustainability efforts around the league. https://www.nba.com/news/nba-green-dr-allen-hershkowitz-leads-sustainability-efforts-around-the-league NBA. (2023). 2021-22 Social Impact Report. https://ak-static.cms.nba.com/wp-content/uploads/sites/144/2023/05/NBA22_ESG_00_Full_FINAL_print-1.pdf	MLB. (2023). MLB Green. https://www.mlb.com/mlb-together/green MLB. (2023). MLB names San Francisco Giants as the recipient of the 2022 "Green Glove Award". https://www.mlb.com/press-release/press-release-mlb-names-san-francisco-giants-as-the-recipient-of-the-2022-green-?t=mlb-press-releases	NHL. (2023). NHL Green. https://www.nhl.com/sv/community/nhl-green/ NHL. (2014). 2014 NHL Sustainability Report. https://ice.nhl.com/green/report/#NHLGreenIntro

Next, we examined sustainability efforts reported by selected teams (Table 1). Overall, most teams (seven out of 12 teams) did not have environmental programs listed on their websites. These include the three NFL teams and teams from New York City- NY Knicks of the NBA, NY Yankees of MLB, and NJ Devils of the NHL. Among the few teams who reported “green” initiatives, their efforts resembled that of their respective leagues- they primarily focused on reducing carbon footprint and waste. To reduce carbon emissions, they planted trees and encouraged fans to take public transit or bike. To reduce waste, they organized recycling, reducing, and reusing programs. It is noteworthy to point out that all NHL teams wear a jersey that is made of 100% recycled polyester, a league-wide initiative toward ending plastic waste. Focusing on food, two teams—Chicago Cubs and LA Kings—have initiatives to source healthy, sustainably produced produce. The LA Kings reported building four organic gardens for healthy produce at local YMCAs whereas the Chicago Cubs reported that they “sourced locally and sustainably produced produce whenever possible”. There is, however, limited information available online about their food programs.

Taken together, initiatives at the team levels are executed largely through forging partnerships with external organizations and/or implementing programs at the stadia (Table 5).

**Table 5:** Sustainability programs implemented by the teams and their respective home stadium.

	NFL			NBA		
**Team**	NY Giants	Chicago Bears	LA Chargers	New York Knicks	Chicago Bulls	Los Angeles Clippers
Team owner	John Mara and Steve Tisch	Virginia Halas McCaskey	Dean Spanos	Madison Square Garden Sports Corp. (MSG Sports)	Jerry Reinsdorf	Steve Ballmer
Carbon emission reduction						Tree planting – Planted 1,000 trees in Inglewood to celebrate the opening of the Clippers new home, Intuit Dome, in 2024.
Waste reduction					Reuse – Partner with community organizations through the "Planters Project" to reuse and repurpose recycled materials – Donated 240 basketballs to 12 schools	
Jersey (sustainable materials)						100% Recycled Polyester. (Nike)
Water conservation						
Sustainable food program						
**Stadium**	MetLife Stadium	Soldier Field	SoFi Stadium	Madison Square Garden	United Center	Crypto.com Arena
Stadium owner	MetLife Stadium Company, LLC	Chicago Park District	Stan Kroenke	Madison Square Garden Entertainment Corp. (MSG Entertainment)	Jerry Reinsdorf and Rocky Wirtz	Anschutz Entertainment Group (AEG)
Carbon reduction	Energy use: – Installed LED lights – Installed solar panels – installed motion sensor lighting Transportation – Encourage fans to use public transportation	Energy use – Replace traditional lighting with LED lights Transportation – Provide 3 free electric vehicle charging stations – Provide bike racks – Encourage fans to take public transit (e.g., #128 Soldier Field Express on gamedays only)	Energy use – Install efficient lighting fixtures, HVAC system, variable speed fans and escalators. Transportation – Encourage alternative transportation- Electric shuttle, LA Metro, etc. – provide 119 bike racks and lockers – Provide 595 clean-air vehicle parking spaces	Energy use – Replace incandescent lighting with LED lights and energy-efficient fluorescents – Installed energy analytic management system to help reduce energy consumption – Applied insulation materials on the roof of the stadium to enhance temperature control and energy efficiency Transportation – Encourage fans to use public transportation – Most cleaning products (e.g., disinfectants and degreasers) are manufactured on-site thus reducing transportation		Energy use – Installed 1,727 solar panels on its rooftop – Installed Bloom Energy fuel cells which generate electricity on-site – Installed LED lights in all offices – Installed LED sports lighting system – Replaced magnetic ballast with electronic ballast – Use time schedules and photocell control for exterior lighting – Use variable speed drives (VSD) on all air handlers and a chiller Transportation – Encourage fans to take public transportation – Provide sufficient bike racks
Waste reduction	Recycle – Provide recycling bins for plastic bottles, aluminium cans – encourage tailgating fans to use reusable utensils and packaging items Reduce – Provide water refilling stations – Donate unused food to reduce waste	Recycle – Recycle cardboard, aluminium, plastic, light bulbs, delivery pallets, batteries, and eyeglasses Reuse – Reuse soil and sod removed from the field for landscaping projects – Reuse and refill cleaning spray bottles and containers	Recycle – Recycle e-waste, aluminium, plastic, cardboard, batteries, ink and toner Reduce – Use recyclable aluminium cups for draft beer – Provide 20 water refilling stations – Donate unused food – Convert used cooking oil to biofuels – Compost landscaping wastes – Use biodigester to reduce food wastes	Recycle – Installed signages and receptacles for recycling programs – Recycle electronics, glass, metal, plastic, paper, cardboard, cooking oil, wood pallets, refrigerant, batteries, lightbulbs, and ballasts. Reduce: – compost on-site as required by New York state law since 2015 – Convert food scrapes to biofuels – Mobile-only ticketing model Procurement policy – toilet paper is made from 100% recycled paper – Most cleaning products are made with 100% recycled plastics or cardboard – Use food packaging items that are from recycled materials or have reduced carbon footprints (e.g., plates made from bamboo or fallen palm leaves, paper containers and cups made of corn oil) – 90% of food packaging items are compostable, reusable or recyclable.		Recycle – Provide receptacles for recyclables Reduce – Removed the use of plastic straw
Water conservation	Water-saving fixtures: – waterless urinals	Water-saving fixtures – water-saving devices in restrooms	Water-saving fixtures – Install low-flow fixtures Reuse – Use the lake in Hollywood Park to capture water and use it for irrigation and aesthetic use	Water-saving fixtures – Installed sensor facets in restrooms – Replaced packing seals with mechanical seals on condenser water – Installed low-flush toilets		Water-saving fixtures – Introduced the BluEco Liquid Crystalline Turbex™ technology, an advanced air-management system that captures water from the air, stores it in water tanks and produces water with high purity while efficiently managing airflow in the stadium. – Waterless urinals
Sustainable food policy	– encourage tailgating fans to prioritize locally sourced foods and organic options		– Implement Responsible Sourcing Policy, including a specific guideline on food and beverage: "1) local (80% within 250 miles), 2) healthy (vegetarian, vegan, gluten-free options), and 3) sustainably sourced food products"			
Human health	– Increased frequency of cleaning with hospital-grade cleaning supplies – Touchless hand sanitizer dispensers throughout the stadium – Upgraded HVAC filtration system for indoor spaces – Fans are asked to stay home if experiencing COVID-19 symptoms	– "If you have a fever, cough, shortness of breath, or have been exposed to someone with these symptoms, please visit us at a later date. " – Cashless payment	– Indoor air quality management	– Cashless payment	– Cashless payment	– "Face coverings are not required, but are strongly recommended for everyone" – "If you’re not feeling well, please stay home!" – "Clean your hands often by washing with soap and water for 20 seconds or using hand sanitizer." – Installed touchless hand sanitizer stations, faucets, soap dispensers, and paper towel dispensers – Cashless transaction – use Atmos Air and BluEco technology to purify the air inside the arena, and MERV-15 air filters which exceed the current standards." – "High-touch surfaces and heavily trafficked areas are continuously disinfected, and all spaces undergo a rigorous disinfection and cleaning after every event."
References	New York Giants. (2023). New York Giants: In The Community. https://www.giants.com/community/ MetLife Stadium. (2023). Stadium Safe. https://www.metlifestadium.com/guest-services/stadiumsafe	Chicago Bears. (2023). Community. https://www.chicagobears.com/community/ U.S. Green Building Council. (2023). Chicago Park District - Soldier Field. https://www.usgbc.org/projects/chicago-park-district-soldier-field	Los Angeles Chargers. (2023). Community. https://www.chargers.com/community/ Sofi Staidum. (2023). Sustainability. https://www.sofistadium.com/sustainability/ Sofi Staidum. (2023). Plan your visit. https://www.sofistadium.com/planyourvisit/	New York Knicks. (2023). Community. https://www.nba.com/knicks/community?icmp=int_web_knicks_comm_parent_nav_220124 Madison Square Garden Entertainment. (2023). Corporate Social Responsibility Report 2022. https://www.msgentertainment.com/wp-content/uploads/2022/11/MSG_2022_CSR_Report.pdf	Chicago Bulls. (2023). Community. https://www.nba.com/bulls/community NBA. (2023). The Bulls’ “Planters Project” Demonstrates the Creative Possibilities of Repurposed Materials. https://www.nba.com/bulls/news/the-bulls-planters-project-demonstrates-the-creative-possibilities-of-repurposed-materials United Center. (2023). Plan your visit. https://www.unitedcenter.com/plan-your-visit/	LA Clippers. (2023). Community. https://www.nba.com/clippers/community Crypto.com Arena. (2023). Environmental Sustainability. https://www.cryptoarena.com/arena-info/environmental-sustainability Crypto.com Arena. (2023). Health and Safety Guidelines. https://www.cryptoarena.com/arena-info/safe AEG. (2019). AEG's 2019 Sustainability Report. https://cms.nhl.bamgrid.com/images/assets/binary/307288540/binary-file/file.pdf

	MLB			NHL		
**Team**	NY Yankees	Chicago Cubs	LA Dodgers	NJ Devils	Chicago Blackhawks	LA Kings
Team owner	Hal Steinbrenner	The Ricketts Family	Guggenheim Baseball Management	Harris Blitzer Sports & Entertainment	Rocky Wirtz	Philip Anschutz and Edward Roski Jr
Carbon emission reduction		Transportation – Provide free bicycle vale services to fans – Encourage fans to take public transit as the stadium is near public transportation hubs Energy use – Installed energy-efficient hot water systems with energy management controls, efficient appliances, and smart lighting and HVAC systems – Sourced construction materials that are harvested and manufactured within 500 miles of the site to reduce transportation emission – Installed green roofs on Hotel Zachary, Gallagher Way and the broadcast building.	Tree planting – To celebrate Earth Day, planted 60 trees at Sycamore Grove Park (2019) – Planted olive trees, palm trees and the native succulent plants of the region in surrounding parks (2017)		Tree planting – Partner with Chicago Gateway Green, a nonprofit organization that helps plant trees in expressways and neighbourhoods.	Transportation – Encourage fans to take public transportation
Waste reduction		Recycle – Provide recycling bins throughout the ballpark – Transport construction debris to recycling centers Reduce – Use compostable food packing and utensils in the ballpark	Reduce – Donate leftover food to Midnight Mission – Donated used furniture from the sales office remodel to Dream Foundation in 2019			Recycle – Recycle bins for e-waste, cans and bottles throughout the arena
Jersey (sustainable materials)				Yes. 100% Recycled Polyester (Adidas Primegreen)	Yes. 100% Recycled Polyester (Adidas Primegreen)	Yes. 100% Recycled Polyester (Adidas Primegreen)
Water conservation						Water credit – Buy water restoration certificates to offset arenas' water usage Education – Hold information sessions for fans on water conservation and recycling
Sustainable food program		-"sourced locally and sustainably produced produce whenever possible"				Garden – Built 4 organic gardens for healthy foods at local YMCAs
**Stadium**	Yankee Stadium	Wrigley Field	Dodger Stadium	Prudential Center	United Center	Crypto.com Arena
Stadium owner	New York City Economic Development Corporation	The Ricketts Family	Guggenheim Baseball Management	Harris Blitzer Sports & Entertainment	Jerry Reinsdorf and Rocky Wirtz	Anschutz Entertainment Group (AEG)
Carbon reduction	Energy use: – Installed light-emitting diode (LED) lights that are 40% more efficient and 50% brighter than previous field lighting. – Installed automation management system to monitor and eliminate inefficient energy use – Architectural design of the public entry allows for natural cooling and ventilation, reducing the use of air conditioning Transportation – Encourage fans to take mass transit to get to the stadium – Provides no public parking garage – Offers high capacity bike-sharing stations around the Stadium – Use trash compactor for waste to reduce the number of garbage trucks needed Offsetting – Offset unavoidable GHG emissions through collaboration with The South Pole Group.	-free bike valet service	Energy use: – Installed new power and lighting energy-efficient systems Transportation – Provide multiple bike rack lockup locations throughout the stadium – Provide free transportation, the Dodger Express, for fans from two locations- Union Station and the South Bay			Energy use – Installed 1,727 solar panels on its rooftop – Installed Bloom Energy fuel cells which generate electricity on-site – Installed LED lights in all offices – Installed LED sports lighting system – Replaced magnetic ballast with electronic ballast – Use time schedules and photocell control for exterior lighting – Use variable speed drives (VSD) on all air handlers and a chiller Transportation – Encourage fans to take public transportation – Provide sufficient bike racks
Waste reduction	Recycle – Recycle cardboard, glass, metal, plastics and paper – Recover and recycle cooking oil to produce biodiesel Reduce – Use of compostable cutlery and food packaging – Compost food waste	Reduce – use compostable food packing and utensils in the ballpark	Reduce – Water Bottle Filling Stations throughout the stadium – Installed hand driers to eliminate paper waste – Use of recyclable drinkable lids and paper straws			Recycle – Provide receptacles for recyclables Reduce – Removed the use of plastic straw
Water conservation	Water-saving fixtures – Installed high-efficiency plumbing fixtures	x	Water-saving fixtures – Installed new water valves, low-flush fixtures, waterless urinals, and automatic faucets to lower water use.			Water-saving fixtures – Introduced the BluEco Liquid Crystalline Turbex™ technology, an advanced air-management system that captures water from the air, stores it in water tanks and produces water with high purity while efficiently managing airflow in the stadium. – Waterless urinals
Sustainable food policy	Garden – Installed a vertical, aeroponic Tower Garden at the stadium to grow fresh produce that is used to make food items served to fans (e.g. salads)					
Human health	-Regular replacement and the use of high-performance filters to improve air quality in the HVAC system COVID-19: – Contactless ticketing – Cashless transaction to reduce contact – Single-use cleaning tools (e.g., mops, wipes) to reduce cross-contamination. – Cleaning protocol based on the WELL Health Safety Rating and Standard – Employees of the stadium will be health screened as appropriate – Updated uniform policies may require staff to wear face coverings	-Cashless payment	-Cashless payment	-Cashless payment	-Cashless payment	-"Face coverings are not required, but are strongly recommended for everyone" – "If you’re not feeling well, please stay home!" – "Clean your hands often by washing with soap and water for 20 seconds or using hand sanitizer." – Installed touchless hand sanitizer stations, faucets, soap dispensers, and paper towel dispensers – Cashless transaction – Use Atmos Air and BluEco technology to purify the air inside the arena, and MERV-15 air filters which exceed the current standards." – "High-touch surfaces and heavily trafficked areas are continuously disinfected, and all spaces undergo a rigorous disinfection and cleaning after every event."
References	NY Yankees. (2023). Yankees In the Community. https://www.mlb.com/yankees/community NY Yankees. (2023). Yankee Stadium Reference Guide. https://www.mlb.com/yankees/ballpark/information	Chicago Cubs. (2023). 2022 Neighborhood Protection Report. https://mktg.mlbstatic.com/cubs/documents/2022_Cubs_Neighborhood_Protection_Plan_Annual_Report.pdf Chicago Cubs. (2023). Wrigley Field. https://www.mlb.com/cubs/ballpark Chicago Cubs. (2023). Cubs in the Community. https://www.mlb.com/cubs/community	LA Dodgers. (2023). Community Relations and External Affairs. https://www.mlb.com/dodgers/community LA Dodgers. (2023). LA Dodgers Community Report. https://www.digitalpublications-mlb.com/157005/157249/186082/Dodgers-2019-Community-Report/?_gl=1*aoga5f*_gcl_au*MTM2ODA3NTg1My4xNzA0ODE2MjYw MLB. (2023). Club Initiatives. https://www.mlb.com/mlb-together/green/club-initiatives	New Jersey Devils. (2023). Community. https://www.nhl.com/devils/community/ Prudential Center. (2023). Arena Guide. https://www.prucenter.com/arena-information	Chicago Blackhawks. (2023). In the Community. https://www.nhl.com/blackhawks/community/ United Center. (2023). Plan your visit. https://www.unitedcenter.com/plan-your-visit/	LA Kings. (2023). Sustainability. https://www.nhl.com/kings/community/kings-care/green AEG. (2019). AEG's 2019 Sustainability Report. https://cms.nhl.bamgrid.com/images/assets/binary/307288540/binary-file/file.pdf Crypto.com Arena. (2023). Environmental Sustainability. https://www.cryptoarena.com/arena-info/environmental-sustainability Crypto.com Arena. (2023). Health and Safety Guidelines. https://www.cryptoarena.com/arena-info/safe

Finally, we examined sustainability efforts at stadia that are home to the selected teams. We found that many of these stadia (eight out of 12 stadiums) are privately owned by the same owner of the team. In such cases, these stadia are less likely to have implemented sustainability efforts. These stadia include the United Center, Wrigley Field, Prudential Center, and the United Center. On the contrary, stadia that are owned by a third party and rented by the team (i.e., Soldier Field, SoFi Stadium, Crypto.com Arena, and Yankee Stadium) have implemented various sustainability efforts.

Among stadia that reported sustainability initiatives, they primarily focus on addressing three aspects: (1) to reduce carbon emission, (2) to reduce waste, and (3) to reduce water use. These are achieved largely through retrofitting. To reduce carbon emissions, most stadia strive to reduce energy use by replacing traditional lighting with LED lights, installing solar panels, and enhancing the efficiency of HVAC systems (e.g., MetLife stadium, Sofi Stadium, Crypto.com Arena). They also encourage alternate transportation by providing bike racks, and bike valet service as well as installing charging ports for electric cars. To reduce waste, they encourage recycling by providing receptacles for a variety of items including e-waste, batteries, cardboard, aluminium, and plastics (e.g., Yankee Stadium, Madison Square Garden, Soldiers Field). They also encourage reducing waste by donating unused food, providing water bottle refilling stations, composting organic wastes, and using compostable food packaging. To conserve water, many stadia have installed automatic faucets, low flush fixtures and waterless urinals.

In addition to addressing environmental health, many stadia have implemented policies to enhance human health considering COVID-19. For example, almost all stadia implemented a cashless payment policy to reduce physical contact. Some also introduced contactless ticketing, improving indoor air quality, increasing cleaning frequency, and asking fans to stay home if they are not well.

Finally, focusing on food, we found that two stadia have food programs in place to promote sustainably produced food and provide healthy food on-site. Interestingly, both stadia are owned by a third party instead of the owner of the team. One stadium, SoFi Stadium (owned by Stan Kroenke) which is home to the LA Chargers (owned by Dean Spanos) has established a sustainable purchasing policy for various goods including food and beverage. The policy states that “buying local (80% within 250 miles), healthy (vegetarian, vegan, gluten-free options) and sustainably sourced food products” is a priority of their organization. It is unclear though how “sustainably sourced” is defined and if meat products are included.

The other stadium that considers healthy food options is Yankee Stadium (owned by New York City Industrial Development Agency), home to the NY Yankees (owned by Hal Steinbrenner). They have installed a vertical garden to grow fresh produce that is used to make food items at the stadium. Existing food programs at these two stadia primarily focused on sourcing produce but overlooked the sourcing of animal-based meat products. Lastly, while it did not report a food program on-site, the MetLife Stadium, home to the NY Giants (which owns 50% of the stadium), posted a reminder online to encourage tailgating fans to prioritize locally sourced food and organic options. Taken together, there are some food programs at stadia that promote the consumption of sustainably sourced foods, but they largely focus on produce only and overlook meat products derived from animal agriculture.

## Discussion

In this content analysis case study, we adopt the One Health approach to demonstrate how the consumption of meat products in sports stadia, derived most likely from excessive use of antibiotics in livestock production in the U.S., threatens sports goals for sustainable development. The production of meat-based products is often interrelated with the rapid emergence of antibiotic-resistant bacteria and environmental contamination, endangering the health and well-being of animals, humans, and the natural environment, and damaging the integrated health of the ecosystem.

We examined whether the four major sports leagues, major leaders in the sustainability movement and consumers of animal-based meat products, have implemented meat sourcing policy that would promote sustainable meat production practices and the reduction of consumption of meat produced with excessive antibiotics, to help safeguard the sustainable health of the ecosystem and contribute to sustainable development.

Results show that existing sustainability efforts by the four major sports leagues, teams, and stadia have primarily focused on reducing carbon emissions and waste. Relatively few efforts focused on food. While some organizations do have food programs to advocate for sustainable food choices (e.g., the MLB, Chicago Cubs, LA Kings, SoFi Stadium and Yankee Stadium), these programs primarily focused on produce and largely overlooked meat products.

We also find that current sustainability initiatives at the team level resembled those of the leagues. This suggests that league-initiated programs may have trickle-down effects. The leagues have the capacity to be conduits of sustainability messaging to promote pro-environmental actions among their teams. It is thus important for the four major leagues to take the lead in establishing environmentally friendly food sourcing initiatives and influence their teams to adopt responsible food consumption and sourcing practices.

Moreover, we found that very few stadia have implemented sustainable food programs. Considering stadia are the key sites of food consumption, it is hence critical for the leagues and teams to work collaboratively to ensure that there are responsible food sourcing policies at stadia to help minimize the ecological impacts of meat consumption during sports events and raise awareness about sustainable food choices among fans. It is also interesting to note that stadia that are owned by a third party are more likely to have sustainability initiatives in place than those that are owned by the owner of the team. For example, the three stadia—United Centre, Wrigley Field, and Prudential Center—did not report sustainability initiatives and they are owned by people who also own the team. This suggests a need to further investigate how stadium ownership may affect sustainability practices at stadia and the underlying mechanisms that could encourage stadium operators to adopt sustainability practices.

Given the far-reaching consequences of antibiotic overuse in livestock production in the U.S., and environmental pollution that is associated with industrialized animal agriculture, there is an urgent need for the leagues, teams, and stadia to adopt meat sourcing policies as part of their sustainability initiatives, which is critical for restoring the One health balance for sustainable development. Meatsourcing policy should include procuring meat products that (1) are raised without antibiotics^1^ and (2) from producers who adopt stringent livestock health management practices (e.g., sanitization, proper manure disposal, humane handling) to maximize animal health and minimize ecological impacts of livestock production and maintaining One Health (148, 149).

While this study urges sport governing bodies to procure meat products produced without the use of antibiotics to enhance planetary sustainability, it should be noted that other scholars and activist organizations have suggested consuming less meat may also contribute to planetary health by improving human health (e.g., lowering risks of cancer) (150, 151) as well as environmental health (e.g., reducing greenhouse gas emission) (16, 152, 153). Hence, in addition to implementing policies to procure meat produced without the use of antibiotics, sports organizations may also consider de-coupling the relationship between sports and meat, providing less-meat, and more plant-rich dishes and plant-based alternatives at sports stadia. This may serve to both raise awareness of the health benefits of eating less meat and more vegetables and to contribute to planetary sustainability.

Collective efforts to promote animal welfare and sustainable meat and food production by the four leagues at the three levels (leagues, teams, and stadia) can have unmatched effects in driving social changes in eradicating the excessive use of antibiotics in livestock farming in the U.S., and working to improve environmental conditions, while advancing sustainable development for three major reasons.

First, sport is a key social platform that can reach millions of people. The sports industry is currently one of the largest economic sectors in the U.S., generating over $552 billion in economic activity every year (154). It wields considerable economic and cultural influences. This is why the United Nations has specifically recognized sport as an effective enabler of social change and an important contributor in advancing various sustainable development goals (155).

Implementing sustainable food and meat policies in the four major sports leagues can help raise public awareness of antibiotic overuse and subsequent environmental degradation in livestock production and promote sustainable food consumption habits among sports fans and beyond. Studies have shown that sustainability initiatives undertaken in sports events can often yield favourable attitudes toward environmental protection and influence pro-environmental behaviours among consumers (23, 156). Meat and food policies at the three levels can likely enhance consumers' understanding of industrialized meat production practices and encourage them to make informed choices by supporting first, meat products produced without the use of antibiotics and, in the future, by adopting less-meat and a more plant-rich diet resulting in positive changes in attitude and consumption behaviors.

Second, collective efforts by the four major sports leagues to source responsibly produced meat and food products can shift the culture of the marketplace by increasing their demand, making them accessible and affordable to consumers. As Chernushenko states “By taking a firm stand in favour of organic food, you [sports organizers] can make a strong statement in favour of healthier farming practices while at the same time bringing down prices by expanding demand” [(118): 191].

Lastly, despite the current lack of antibiotic regulations on the meat industry in the U.S., introducing sustainable meat and food policies at the four major sports leagues will help garner public support and awareness on the issues of antibiotic overuse in livestock production, and water, soil and air pollution. This potentially could influence institutional changes, such as stricter regulations to protect animal welfare and bans to curb the excessive use of antibiotics in livestock farming and practices that cause ecosystem destruction. Indeed, the EU, through its antibiotic ban, has demonstrated that it is both feasible and advisable to raise livestock without resorting to antibiotics when proper management practices (e.g., animal welfare practices, stringent sanitization) are implemented. Institutional changes will be imperative to help tackle the public health crisis caused by the emergence of antibiotic resistance bacteria and pollution and their threats to sustainable development.

The findings of this study contribute to the literature by showing the current state of sustainability efforts across the four major sports leagues, their teams, and stadia, and demonstrating how meat and food sourcing is a missing piece in current sustainability initiatives aimed at ensuring planetary sustainability in the sport sector. Results can also inform international and national sports governing agencies that meat and food sourcing policies should be considered in sustainability planning and policy recommendations to encourage the consumption of responsibly produced meat and food products and boycott industrialized animal agricultural production practices that damage planetary health.

A limitation of this study is that the data is collected from the official websites of the leagues, teams, and stadia. There is a possibility that data included in the analysis may differ from actual practices given that organizations may not have published the most up-to-date information online. Also, we included twelve top-performing teams across the four leagues in the three most populated cities in the U.S. in our analysis, therefore future research should include more teams to draw more comprehensive results and engage surveys and interviews with sports procurement managers and fans to determine the desire for change in sports.

## Conclusion

A growing body of evidence suggests that industrial animal agricultural production practices with the excessive use of antibiotics pose significant harm to animals, humans, and the environment, threatening the sustainable health of the planet. However, we found that current sustainability initiatives implemented by the four major sports leagues, their teams, and stadia primarily focused on carbon emissions and waste reduction, with limited attention to food sourcing policies aimed at mitigating the ecological impact of meat consumption. Given the growing demand of meat products at sports events, there is an urgent need for the four major sports leagues to leverage their unparalleled influence in promoting responsible meat production and consumption by incorporating sustainable meat sourcing policies in their sustainability initiatives. The findings of this study can also inform international sports organizations of the importance of integrating meat sourcing policies into sustainability planning to encourage sustainable dietary practices in the sports sector, thereby safeguarding the integrated health of the planet.

## Data Availability

The original contributions presented in the study are included in the article/Supplementary Material, further inquiries can be directed to the corresponding authors.

## Footnotes

^1^It should be noted that labels such as “antibiotic-free” can be misleading because all meat is technically “antibiotic-free” as required by federal regulations which restrict that all meat sold at stores should not contain antibiotics exceeding the acceptable legal limits (146, 147). “Antibiotic-free”, therefore, does not imply that no antibiotics were used in livestock production. To ensure antibiotics are not used in livestock production, consumers should look for labels such as “no antibiotics administered”, “raised without antibiotics”, or “not treated with antibiotics”. Yet, they should also buy from trusted, reputable suppliers as some of these labels have been found to be deceiving as well (61).
